# Increased GH/IGF-I Axis Activity Relates to Lower Hepatic Lipids and Phosphor Metabolism

**DOI:** 10.1210/clinem/dgad206

**Published:** 2023-04-27

**Authors:** Paul Fellinger, Hannes Beiglböck, Georg Semmler, Lorenz Pfleger, Sabina Smajis, Clemens Baumgartner, Martin Gajdosik, Rodrig Marculescu, Greisa Vila, Yvonne Winhofer, Thomas Scherer, Michael Trauner, Alexandra Kautzky-Willer, Martin Krssak, Michael Krebs, Peter Wolf

**Affiliations:** Division of Endocrinology and Metabolism, Department of Internal Medicine III, Medical University of Vienna, 1090 Vienna, Austria; Division of Endocrinology and Metabolism, Department of Internal Medicine III, Medical University of Vienna, 1090 Vienna, Austria; Division of Gastroenterology and Hepatology, Department of Internal Medicine III, Medical University of Vienna, 1090 Vienna, Austria; Division of Endocrinology and Metabolism, Department of Internal Medicine III, Medical University of Vienna, 1090 Vienna, Austria; Centre of Excellence-High Field MR, Department of Biomedical Imaging and Image-guided Therapy, Medical University of Vienna, 1090 Vienna, Austria; Division of Endocrinology and Metabolism, Department of Internal Medicine III, Medical University of Vienna, 1090 Vienna, Austria; Division of Endocrinology and Metabolism, Department of Internal Medicine III, Medical University of Vienna, 1090 Vienna, Austria; Centre of Excellence-High Field MR, Department of Biomedical Imaging and Image-guided Therapy, Medical University of Vienna, 1090 Vienna, Austria; Department of Laboratory Medicine, Medical University of Vienna, 1090 Vienna, Austria; Division of Endocrinology and Metabolism, Department of Internal Medicine III, Medical University of Vienna, 1090 Vienna, Austria; Division of Endocrinology and Metabolism, Department of Internal Medicine III, Medical University of Vienna, 1090 Vienna, Austria; Division of Endocrinology and Metabolism, Department of Internal Medicine III, Medical University of Vienna, 1090 Vienna, Austria; Division of Gastroenterology and Hepatology, Department of Internal Medicine III, Medical University of Vienna, 1090 Vienna, Austria; Division of Endocrinology and Metabolism, Department of Internal Medicine III, Medical University of Vienna, 1090 Vienna, Austria; Division of Endocrinology and Metabolism, Department of Internal Medicine III, Medical University of Vienna, 1090 Vienna, Austria; Centre of Excellence-High Field MR, Department of Biomedical Imaging and Image-guided Therapy, Medical University of Vienna, 1090 Vienna, Austria; Division of Endocrinology and Metabolism, Department of Internal Medicine III, Medical University of Vienna, 1090 Vienna, Austria; Division of Endocrinology and Metabolism, Department of Internal Medicine III, Medical University of Vienna, 1090 Vienna, Austria

**Keywords:** hepatic steatosis, growth hormone, growth hormone deficiency, IGF-I, NAFLD, ^1^H/^31^P magnetic resonance spectroscopy

## Abstract

**Context:**

Non-alcoholic fatty liver disease (NAFLD) is a leading causes of liver-related morbidity and mortality. While data on acromegaly, a state of chronic growth hormone (GH)/insulin-like growth factor I (IGF-I) excess, suggest an inverse relationship with intrahepatic lipid (IHL) content, less is known about the impact of the GH/IGF-I axis on IHL, lipid composition, and phosphor metabolites in individuals without disorders of GH secretion.

**Objective:**

The aim was to investigate the relation between activity of the GH/IGF-I axis and IHL content and phosphor metabolism.

**Methods:**

We performed a cross-sectional study in 59 otherwise metabolically healthy individuals (30 females), of which 16 met the criteria of NAFLD with IHL of ≥5.6%. The GH/IGF-I axis was evaluated in a fasting state and during an oral glucose tolerance test (OGTT). Insulin sensitivity was estimated by validated indices. IHL, lipid composition (unsaturation index), and phosphate metabolites were analyzed by using ^1^H/^31^P magnetic resonance spectroscopy.

**Results:**

In the overall cohort (40.6 ± 15 years; body mass index: 24.5 ± 3 kg/m^2^; IGF-I: 68.0 ± 17% upper limit of normal), fasting GH (R = −0.31; *P* = .02), GH during oral glucose tolerance test (R = −0.51; *P* < .01), and IGF-I (R = −0.28; *P* = .03) inversely correlated with IHL. GH levels during OGTT were significantly lower in NAFLD than in controls (47.7 [22; 143] ng/mL/min vs 16.8 [7; 32] ng/mL/min; *P* = .003). GH/IGF-I axis activity correlated with lipid composition and with phosphor metabolites. In multiple regression analysis, the GH/IGF-I axis activity was a strong predictor for IHL and lipid composition independent from insulin sensitivity.

**Conclusion:**

GH/IGF-I axis activity impacts hepatic lipid and phosphate metabolism in individuals without disorders in GH secretion. Lower GH axis activity is associated with higher IHL and an unfavorable lipid composition, probably mediated by changes in hepatic energy metabolism.

Nonalcoholic fatty liver disease (NAFLD) is considered to be the hepatic manifestation of the metabolic syndrome and is among the leading causes of liver-related morbidity and mortality. Despite its high prevalence, pharmacological therapy is missing (1). Recent studies in patients suffering from acromegaly, a clinical syndrome caused by increased concentrations of growth hormone (GH) and insulin-like growth factor 1 (IGF-I), reported very low levels of intrahepatic lipids (IHLs) compared with healthy controls, even though they present with pronounced insulin resistance (2, 3). On the contrary, decreased levels of GH/IGF-I promote the development of NAFLD, and supplementation of recombinant GH in individuals with GH deficiency reversed hepatic steatosis (4, 5). However, despite these convincing antisteatotic effects of GH/IGF-I in patients with disorders of GH secretion, only limited evidence exists in individuals with normal pituitary function.

Previous studies addressing the antisteatotic effects of GH/IGF-I have important methodological limitations. Pulsatile secretion of GH necessitates dynamic testing during an oral glucose tolerance test (OGTT), whereas single sampling of fasting GH might not be sufficient (6). Most studies that investigated the associations between the GH/IGF-I axis and IHL accumulation therefore focused on singular IGF-I measurements (7). However, dynamic GH measurements might improve characterization of the metabolic status (8). Furthermore, prior studies were mainly based on the use of ultrasound to determine NAFLD. The sensitivity of this method is suboptimal, especially when hepatic fat infiltration is low (9).

The current gold standard for radiologic quantification of IHL accumulation is the use of proton based magnetic resonance spectroscopy (^1^H-MRS), which has repeatedly shown to be superior to ultrasound, especially when hepatic lipid content is in the lower range (10-14) The use of ^1^H-MRS not only allows the precise quantification of intrahepatic fat, but also enables one to assess hepatic lipid composition (15, 16). This gives an insight into de novo lipogenesis and hepatic insulin resistance, which is characterized by a higher lipid saturation fraction. In addition, the combination of ^1^H- and ^31^P-MRS enables quantification of energy-rich phosphates, which are surrogates for hepatic energy metabolism (17, 18). Using ^31^P-MRS, we recently showed that the low levels of IHL in patients with acromegaly were associated with an increased adenosine triphosphate (ATP) synthesis rate (19).

Thus, the aim of this study was to evaluate the impact of the GH/IGF-I axis on hepatic lipid and phosphor metabolism in a cohort of otherwise metabolically healthy individuals without disorders of GH secretion using ^1^H/^31^P-MRS of the liver.

## Materials and Methods

### Study Characteristics

This was a cross-sectional analysis of a cohort of metabolically healthy individuals from 4 previously published studies (19-22) performed at the Division of Endocrinology and Metabolism, Department of Internal Medicine III, Medical University of Vienna. Each study was approved by the local ethics committee (EC-Numbers: 1022/2013, 2039/2013, 1128/2013, 1226/2015, trial registration: NCT02115906; NCT02558270; NCT02075164) and written informed consent was obtained from all participants prior to study related activities.

### Subjects

All subjects were between 18 and 75 years of age, presenting with IGF-I concentrations within the age- and sex-specific limits of normal. The following exclusion criteria were applied: intake of medication affecting glucose or lipid metabolism; HbA1c >6.0%; obesity class 2 or higher (BMI > 35 kg/m^2^); history of liver or renal disorders; risk for advanced hepatic fibrosis as assessed by fibrosis-4 (FIB-4) score >2.67); any acute inflammatory disease within 2 weeks prior to the study; pregnancy and nursing; and any magnetic resonance contraindications including claustrophobia and metal devices in or on the subject's body.

Prior to study participation, all subjects underwent a detailed physical examination and were screened for any acute or chronic metabolic disease. Anthropometric characteristics and blood pressure were assessed systematically. All measurements were carried out after an overnight fast of at least 8 hours. Participants were asked to refrain from physical training and instructed to ingest an isocaloric diet for 3 days prior to the study day. During the standardized 75 g OGTT over 120 minutes, glucose, insulin, c-peptide, and GH were measured at baseline as well as every 30 minutes thereafter. In female participants, a pregnancy test was performed if they were of childbearing age.

### Laboratory Parameters

Laboratory parameters were analyzed using routine laboratory methods at the ISO certified department of Laboratory Medicine of the Medical University of Vienna (kimcl.at). IGF-I concentrations were measured using a chemiluminescence immunoassay on the Liaison autoanalyzer (DiaSorin, Saluggia, Italy, RRID:AB_2928957) and are given as absolute values or as percentages above the assay specific upper limit of normal (ULN) for sex and age. GH concentrations were measured by using the Cobas Human Growth Hormone electrochemiluminescence immunoassay by Roche (Roche Diagnostics, Germany, RRID:AB_2883977). Insulin resistance was estimated by using the Homeostasis Model Assessment of Insulin Resistance (HOMA-IR) and Oral Glucose Insulin Sensitivity (OGIS) as previously described (23, 24). FIB-4 was used as the risk score for hepatic fibrosis and calculated as reported by Sterling et al (25).

### Magnetic Resonance Spectroscopy Measurements

^1^H/^31^P-MRS of the liver was performed on a 7.0-T whole-body magnetic resonance system (Magnetom, Siemens) using 1 of 2 double-tuned (^1^H/^31^P) surface coils (both Rapid Biomedical Ltd), with diameters of 10 and 14 cm (^31^P channel), as previously published (16-19). Phosphor measurements are given as relative to total phosphorous signal. IHL was measured using localized single-voxel GUSTEAU sequence and then calculated from the ratio of summed area of fat to that of water plus fat, with all signals corrected for both T1 and T2 (16). For further subgroup analysis, individuals with IHL ≥5.6% were defined as NAFLD (10). Hepatic lipid composition was assessed by calculating lipid unsaturation index (UI) as the fraction of unsaturated lipids of the total hepatic lipid signal (16). The hepatic ATP synthesis rate (kATP) in the liver was measured by using the ^31^P saturation transfer technique as an indicator of hepatic energy metabolism (17). In addition, basal values of phosphorus-containing metabolites, inorganic phosphate (Pi), nicotinamide adenine dinucleotide phosphate (NADPH), γ-ATP, uridine diphosphate glucose (UDPG), phosphomonoesters (PMEs), phosphoethanolamine, phosphocholine, and phosphodiesters (PDEs), as a sum of glycerophosphoethanolamine and glycerophosphocholine (GPC), were measured as previously reported and validated (19, 26, 27).

### Statistical Analysis

**Figure 1.**
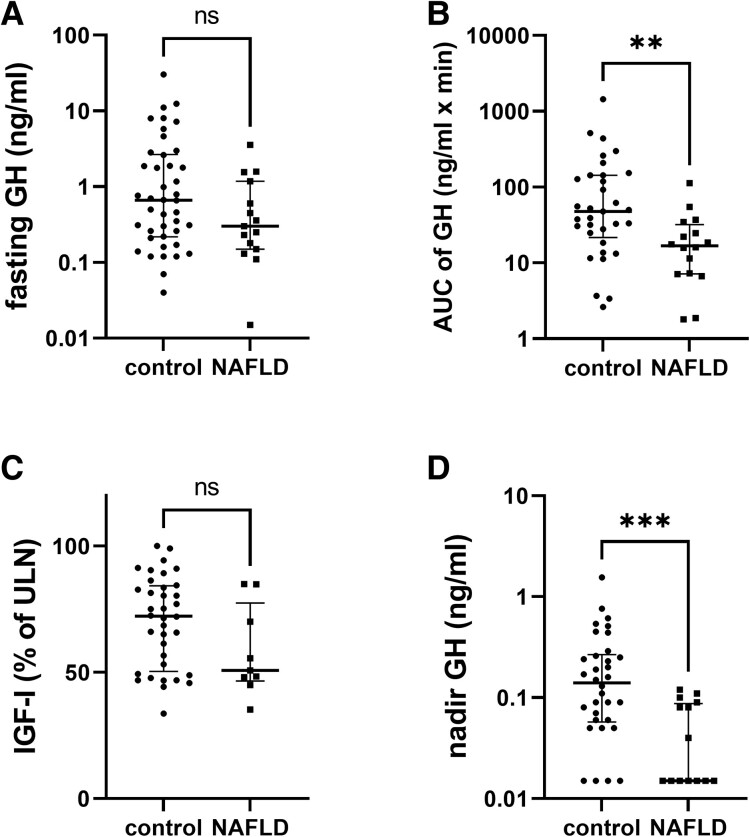
Comparison of fasting GH, dynamic GH concentrations during OGTT (AUC of GH), and IGF-I, and in patients with NAFLD and controls. AUC, area under the curve; GH, growth hormone; IGF-I, insulin like growth factor I; NAFLD, nonalcoholic fatty liver disease; ns, not significant. **P < .01, ***P < .001.

**Figure 2.**
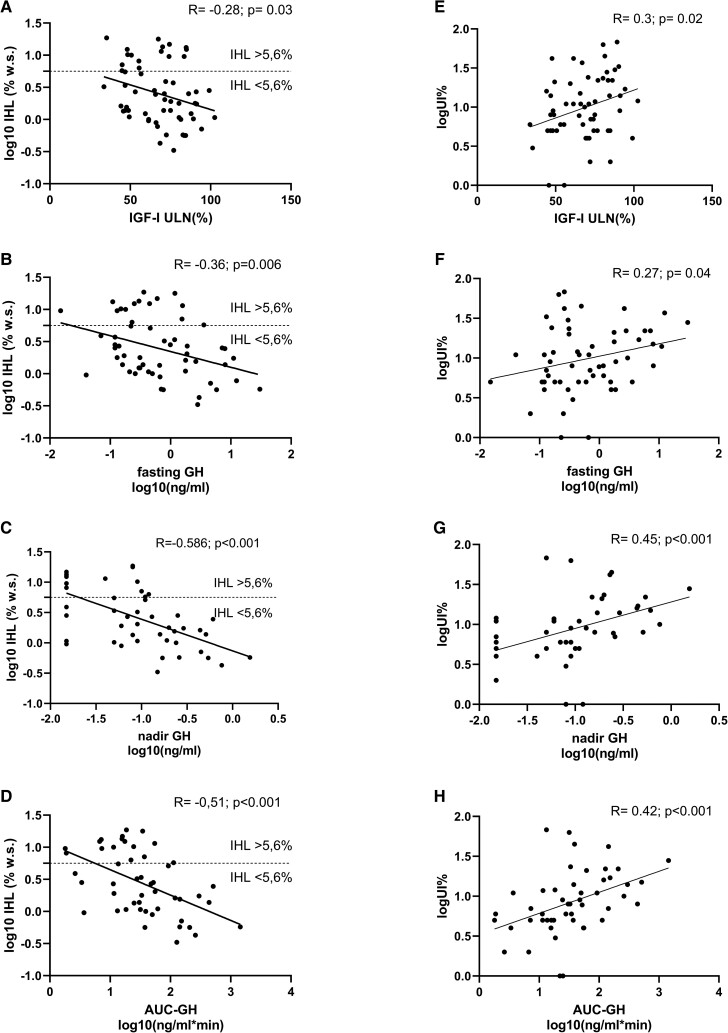
Correlation analysis of parameters of the GH/IGF-I axis and IHL, and UI. AUC, area under the curve; GH, growth hormone; IGF-I, insulin-like growth factor I; IHL, intrahepatic lipid; NAFLD, nonalcoholic fatty liver disease; UI, unsaturation index.

All data are presented as mean **±** SD or as median with interquartile ranges where applicable due to a skewed distribution of data. Area under the curve (AUC) was calculated using the trapezoidal method. The lowest measured GH value within the 2 hour OGTT was taken as nadir GH. For further analysis, all individuals were subdivided into subgroups with IHL of ≥5.6% (NAFLD) and <5.6% (control). Furthermore, we subdivided the control group into 2 groups with low normal and high normal IHL of equal size. Statistical differences between 2 groups were calculated using a 2-sided Student t -test or Mann–Whitney U test, as applicable. Non-normally distributed parameters were log-transformed to achieve normal distribution prior to statistical analysis (Figs. 1 and 2). Relationships between variables were determined by Pearson's correlation coefficient. As this study had an explorative retrospective design, we did not perform a multiple comparison correction. To assess the association between age, BMI, OGIS, IGF-ULN, nadir GH, and hepatic lipid content a linear regression model analysis was performed. GH nadir was chosen as a parameter as it had higher correlation coefficients to IHL than fasting GH and AUC of GH. Data were analyzed as observed and no imputations were used to replace missing data. The limit for statistical significance was defined as **α** < .05. Statistical analyses were performed using GraphPad Prism version 9.4 (GraphPad Software, San Diego, CA, USA).

## Results

### Subjects’ Characteristics

In total, we analyzed 59 individuals, comprising 30 women and 29 men, matching our inclusion criteria. IHL was ≥5.6% in 16 subjects (27.1%). FIB-4 levels were below 2.67 in all participants.

In women, fasting GH (1.17 [0.335; 3.795] vs 0.240 [0.130; 0.7375]; *P* = .0003), AUC of GH (61.80 [27.90; 152.6] vs 15.9 [7.0; 37.2] *P* ≤ .0001), and nadir GH (0.19 [0.08; 0.4] vs 0.06 [0.02; 0.1] *P* < .001) were significantly higher than in men, whereas there was no difference between men and women regarding IGF-I. IHL was higher in men (2.788 [1.370;9.485] vs 1.58 [0.935; 3.493] *P* = .0396) and the UI was lower in men than in women (6.0 [5.0;11.38] vs 14.50 [6.75; 22.35]; *P* = .0165).

Three subjects had a fasting glucose between 100 and 105 mg/dL and 2 subjects had glucose values >140 mg/dL after 120 minutes in the OGTT (148, and 160 mg/dL, respectively) indicative for prediabetes.

Further details regarding baseline characteristics of the whole group are given in Table 1 (see also Table S1 (28)).

**Table 1.** Baseline characteristics of all study participants

Parameter	n = 59Mean ± SD; median (IQR)	Control (n = 43)Mean ± SD median (IQR)	NAFLD (n = 16)Mean ± SD median (IQR)	*P* value (control vs NAFLD)
Clinical/laboratory parameters				
Age (y)	40.64 ± 15.17	39.48 ± 16.12	43.77 ± 12.15	.34
Sex (M/F)	29/30; 49% vs 51%	17/26	12/4	.02
BMI (kg/m^2^)	24.53 ± 3.51	23.24 ± 3.09	27.82 ± 2.41	<.0001
Fasting glucose (mg/dL)	85.27 ± 7.87	83.67 ± 7.41	89.56 ± 7.66	.0094
Fasting GH (ng/mL)	0.46 (0.195; 1.83)	0.665 (0.2175; 2.670)	0.30 (0.15; 1.18)	.12
Glucose AUC (mg/dL × min)	13 830 ± 2738	13 363 ± 2576	14 822 ± 2891	.08
GH AUC (ng/mL × min)	32.70 (13.43; 102.4)	47.70 (21.64;143.0)	16.77 (7.163;32.03)	.0029
Nadir GH (ng/mL)	0.09 (0.015;0.21)	0.14 (0.058; 0.268)	0.015 (0.015;0.09)	.0005
IGF-I (% ULN)	68.02 ± 17.03	70.27 ± 17.03	62.0 ± 16.02	.0976
HbA1C (%)	5.19 ± 0.32	5.17 ± 0.34	5.22 ± 0.27	.62
FIB-4	0.84 ± 0.50	0.81 ± 0.48	0.92 ± 0.55	.4939
HOMA-IR	1.7 ± 0.9	1.268 (0.9360; 2.092)	2162 (1526; 3010)	.0064
OGIS	455.4 ± 75.09	478.3 ± 62.34	406.5 ± 78.73	.0022
^1^H/^31^P-MRS				
IHL (% ws)	4.38 ± 4.78; 2.02 (1.07; 6.26)	1.808 ± 1.213	11.30 ± 3.766	<.0001
UI (% IHL)	14.71 ± 14.46; 9.0 (5.0; 21.0)	14 (8;23.2)	5 (4; 5.8)	<.0001
kAtp (*−1)	0.22 ± 0.11	0.22 ± 0.1065	0.193 ± 0.101	.35
γ-ATP (%TP)	14.61 ± 1.817	14.62 ± 1.48	14.60 ± 2.57	.974
Pi (%TP)	8.483 ± 1.321	8.74 ± 1.19	7.81 ± 1.45	.015
UDPG (%TP)	3.535 ± 1.384	3.79 ± 1.32	2.87 ± 1.37	.022
NADPH (%TP)	5.787 ± 1.78	6.09 ± 1.52	4.99 ± 2.19	.035
PME (PC + PE) (%TP)	15.16 ± 2.949	15.74 ± 2.72	13.61 ± 3.06	.012
PDE (GPC + GPE) (%TP)	36.31 ± 6.37	34.74 ± 5.18	40.41 ± 7.50	.002

Abbreviations: ATP, adenosine triphosphate; AUC, area under the curve; BMI, body mass index; FIB-4, fibrosis-4 score; F/M, female/male; GH, growth hormone; GPC, glycerophosphocholine; GPE, glycerophosphoethanolamine; HOMA-IR, Homeostasis Model Assessment of Insulin Resistance; IGF-I, insulin-like growth factor 1; IHL, intrahepatic lipid content; IQR, interquartile range; kATP, ATP synthase activity; MRS, magnetic resonance imaging; NADPH, nicotinamide adenine dinucleotide phosphate; NAFLD, nonalcoholic fatty liver disease; OGIS, Oral Glucose Index Sensitivity; PC, phosphocholine; PDE, phosphodiester; PE, phosphoethanolamine; Pi, inorganic phosphate; PME, phosphomonoester; TP, total phosphor; UDPG, uridine diphosphate glucose; UI, unsaturation index; ULN, Upper limit of normal.

### Differences Between NAFLD and Controls

In a second step, subjects with IHL <5.6% and IHL ≥5.6% were compared (control vs NAFLD, Table 1). The 2 groups were comparable regarding age, but male sex was more prevalent in the NAFLD group. As expected, BMI was significantly higher in the NAFLD group. HbA1c was not different between the groups. Fasting and dynamic measurements of glucose, insulin, and c-peptide, as well as HOMA-IR and OGIS, were significantly higher in the NAFLD group. Importantly, individuals with NAFLD had lower levels of GH, IGF-I, nadir GH, and GH-AUC; however, only the difference of nadir GH and GH-AUC reached statistical significance. Individual values between the groups are illustrated in Fig. 1.

With regards to parameters of MRS, the ratio of unsaturated to saturated lipids was significantly lower in individuals with NAFLD. Furthermore, significant differences in relative levels of PMEs and PDEs were observed. While GPC, a PDE, was significantly higher in the NAFLD group, PMEs were significantly lower in NAFLD. UDPG and NADPH were significantly different between the groups. In addition, the ratio of phosphoethanolamine, PME, GPC, and NADPH in relation to the sum of PME and PDE were significantly different between control and NAFLD. The ratio between PME and PDE was significantly higher in the control group than in the NAFLD group.

All data of the comparison between the control and the NAFLD group are given in detail in Table 1 (see also Table S1 (28)).

### Correlation Analysis of the GH/IGF-I Axis and MRS/Laboratory Parameters

In the total cohort, fasting GH (R = −0,31; *P* = .02), nadir GH (R = −0.51; *P* ≤ .01) AUC of GH (R = −0.51; *P* < .01) and IGF-I (R = −0.28; *P* = .03) inversely correlated with IHL (see Table 2 and Fig. 2).

**Table 2.** Correlations between fasting GH, nadir GH, AUC-GH, IGF-I ULN, and other clinical and MRS parameters across the entire cohort

	Fasting GH		Nadir GH		AUC GH		IGF-I ULN	
Clinical parameters	R	*P* value	R	*P* value	R	*P* value	R	*P* value
Age	−0.27	.04	−0.174	.227	−0.16	.27	−0.38	.00
BMI	−0.16	.23	−0.350	.013	−0.39	.01	−0.15	.24
Glucose metabolism								
HbA1c	−0.20	.15	−0.145	.317	−0.12	.40	−0.09	.51
OGIS	0.10	.51	0.254	.097	0.30	.05	0.13	.39
HOMA-IR	−0.07	.60	−0.281	.048	−0.46	<.001	−0.03	.82
^1^H-MRS								
log UI	0.27	.04	0.45	<.002	0.42	<.001	0.30	.02
log_IHL	−0.31	.02	−0.59	<.001	−0.51	<.001	−0.28	.03
^31^P-MRS								
kATP	−0.01	.95	−0.030	.839	0.19	.21	0.00	.97
γ-ATP	−0.22	.10	−0.261	.070	−0.28	.05	−0.10	.44
**α**-ATP	0.14	.30	0.048	.743	−0.07	.64	−0.05	.73
Pi	0.13	.32	0.080	.585	0.02	.87	0.08	.55
UDPG	0.33	.01	0.328	.021	0.35	.01	0.25	.06
NADPH	0.14	.31	0.110	.451	0.30	.04	−0.17	.19
PtdCh	0.13	.33	0.201	.165	0.27	.06	−0.17	.20
PC	0.22	.11	0.113	.440	0.15	.31	0.23	.09
PE	−0.23	.09	−0.099	.497	0.19	.19	−0.20	.13
PME (PC + PE)	−0.04	.77	−0.007	.960	0.30	.04	−0.02	.91
GPC	−0.23	.09	−0.238	.100	−0.41	<.001	0.04	.79
GPE	0.11	.42	0.168	.248	0.13	.38	0.07	.62
PDE (GPC + GPE)	−0.13	.35	−0.088	.545	−0.24	.10	0.06	.64
PE/(PME + PDE)	−0.21	.13	−0.087	.551	0.22	.13	−0.20	.12
GPC/(PME + PDE)	−0.23	.09	−0.240	.097	−0.46	<.001	−0.03	.85
PME/PDE	0.00	1.00	0.012	.937	0.26	.08	−0.02	.90
NADPH/(PME + PDE)	0.19	.15	0.153	.294	0.33	.02	−0.15	.26
PE/γ-ATP	−0.18	.19	−0.041	.781	0.21	.15	−0.19	.16
PME/γ-ATP	0.08	.57	0.130	.372	0.39	.01	0.01	.96

Abbreviations: ATP, adenosine triphosphate; AUC, area under the curve; BMI, body mass index; FIB-4, Fibrosis-4 score; GH, growth hormone; GPC, glycerophosphocholine; GPE, glycerophosphoethanolamine; HOMA-IR, Homeostasis Model Assessment of Insulin Resistance; IGF-I, insulin-like growth factor 1; IHL, intrahepatic lipid content; kATP, ATP synthase activity; MRS, magnetic resonance spectroscopy; NADPH, nicotinamide adenine dinucleotide phosphate; OGIS, Oral Glucose Index Sensitivity; PC, phosphocholine; PDE, phosphodiester; PE, phosphoethanolamine; Pi, inorganic phosphate; PME, phosphomonoester; PtdCh, phosphatidylcholine; TP, total phosphor; UDPG, uridine diphosphate glucose; UI, unsaturation index; ULN, upper limit of normal.

**Figure 3.**
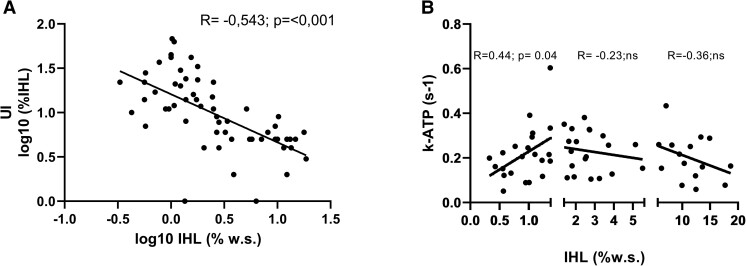
Correlation analysis of IHL and UI, as well as between kATP and IHL. IHL, intrahepatic lipids; UI, unsaturation index.

Interestingly, we did not observe any association between parameters of the GH/IGF-I axis and kATP in the total cohort. Furthermore, there was no significant correlation between kATP and IHL of all observations (see Fig. 2). However, when analyzing individuals in the lower and higher half of IHL separately, a significant positive correlation between kATP and IHL (r = 0.4381; *P* = .0414) was found in individuals in the group with low normal IHL (IHL ≤1.37%). On the other hand, in individuals with high normal lipid content as well as with NAFLD, a negative, but insignificant, association of IHL and kATP was observed (see Fig. 3B). Of note, there were no significant associations between GH or IGF-I and kATP in any subgroup of IHL content (low normal, high normal, or NAFLD).

All parameters of the GH/IGF-I axis correlated significantly with UI. With increasing activity of the GH/IGF-I axis the ratio of unsaturated lipid was increasing (see Table 2 and Fig. 2).

Furthermore, a strong inverse correlation between IHL and UI could be observed in the whole cohort (r = −0.62, *P* ≤ .0001) (see Fig. 3A).

The phosphor metabolites γ-ATP, GPC, UDPG, NADPH, and PME as well as the ratios between GPC/(PME + PDE), NADPH/(PME + PDE), as well as PME/γ-ATP correlated significantly with GH-AUC. While a higher GH-AUC was related to higher γ-ATP, GPC, NADPH/(PME + PDE), as well as PME/γ-ATP, it was inversely related to UDPG, NADPH, PME, and GPC/(PME + PDE). UDPG was the only phosphor metabolite that correlated significantly with fasting GH and with nadir GH.

Details of all correlation analyses are reported in Table 2.

### GH/IGF-I Axis Is Independently Associated With IHL

To evaluate the factors influencing IHL, we performed a linear regression analysis using clinical parameters as well as GH/IGF-I axis parameters and estimates for insulin sensitivity. BMI, IGF-I ULN, and the nadir GH were independent predictors for IHL. In the test for multicollinearity, the variance inflating factor for all variables was between 1.2 and 1.7, suggesting no evidence for multicollinearity (see Table 3).

**Table 3.** Multiple linear regression analysis for the prediction of IHL (Model R^2^ 0.632)

Variable	Estimate	95% CI	F	*P* value
Sex	−0.11	−0.3554/0.1329	0.8579	NS
Age	0.002	−0.006/0.011	0.035	NS
BMI	0.043	0.0073/0.078	6.036	.019
IGF-ULN	−0.766	−1.42/−0.113	5.678	.023
OGIS	−0.001	−0.0027/0.001	1.430	NS
Nadir GH	−0.43	−0.675/−0.189	13.09	.001

Abbreviations: BMI, body mass index; GH, growth hormone; IGF, insulin-like growth factor; IHL, intrahepatic lipid; NS, not significant; OGIS, Oral Glucose Insulin Sensitivity; ULN, upper limit of normal.

In the linear regression analysis for the prediction of UI, nadir GH and IGF-1 were still significant predictors of UI, independent of factors like BMI, which was 1 of the strongest predictors regarding IHL (see Table 4).

**Table 4.** Multiple linear regression analysis for the prediction of UI (model R^2^ 0.332)

Variable	Estimate	95% CI	F	*P* value
Sex	−0.143	−0.416/0.1305	1.128	NS
Age	−9.241 **×** 10^−6^	−0.0092/0009	4 **×** 10^−6^	NS
BMI	−0.00237	−0.042/0.037	0.015	NS
IGF-ULN	0.733	0.0014/1.47	4.145	<.05
OGIS	−4.87 **×** 10^−5^	−0.00196/0.0019	0.0027	NS
Nadir GH	0.2754	0.0035/0.547	4.238	<.05

Abbreviations: BMI, body mass index; GH, growth hormone; IGF, insulin-like growth factor; NS, not significant; OGIS, Oral Glucose Insulin Sensitivity; UI, unsaturation index; ULN, upper limit of normal.

## Discussion

In this cross-sectional study we demonstrate that the GH/IGF-I axis might play an important role in the regulation of hepatic lipid metabolism in individuals without disorders of GH secretion. By combining high-resolution ^1^H/^31^P-MRS with dynamic measurements of GH secretion during the OGTT, we show that GH/IGF-I axis activity is reduced in otherwise metabolically healthy individuals with NAFLD and that GH and IGF-I are both independent predictors of IHL. Furthermore, we found that higher GH activity is associated with a more favorable hepatic lipid composition and changes in hepatic phosphate metabolites.

GH is a strong stimulator of lipolysis in adipose tissue. Therefore acromegaly is a unique condition of severe insulin resistance in the presence of low amounts of whole-body and ectopic adipose tissue (29-31). Inversely, GH deficiency is associated with a higher incidence of NAFLD (3). These findings are suggestive for direct antisteatotic effects of the GH/IGF-I axis in the liver.

Our results extend the findings of a previous study in an independent cohort (32). In line with our results this study showed that GH, but not IGF-I was significantly lower in NAFLD patients (32). In contrast to this recently published study, we not only measured lipid content in the liver but also determined lipid saturation as well as phosphor metabolites by combining ^1^H as well as ^31^P-MRS at high field strengths (32).

When comparing individuals with normal IHL with those with IHL ≥5.6%, nadir GH as well as AUC of GH during OGTT, but not fasting GH and IGF-1, was higher in subjects with normal IHL. As expected, BMI and insulin resistance indices such as HOMA-IR and OGIS were higher in individuals with NAFLD. As obesity and insulin resistance per se have an impact on GH secretion, one might assume that the observed differences in GH/IGF-I axis activity in NAFLD were due to higher BMI and worse glycemic control. However, in multiple regression analysis, IGF-I and nadir GH both remained strong independent predictors for IHL.

We furthermore observed a positive association of the GH/IGF-I axis with UI, the fraction of unsaturated lipids in the liver. UI is associated with de novo lipogenesis and hepatic insulin resistance in patients with NAFLD (15). Increased IHL is associated with a relative reduction of unsaturated lipids, which is consistent with previous publications (33). This suggests that a more active GH/IGF-I axis within the physiological range might lead to a metabolically favorable hepatic lipid profile.

With regards to hepatic energy metabolism, no association between GH/IGF-I axis and the ATP synthesis rate kATP could be found in the whole cohort. Furthermore, kATP was comparable between subjects with and without NAFLD. This is in contrast to our previous observations in patients with acromegaly, where we clearly showed that the kATP is increased comparison with healthy individuals, probably contributing to the very low hepatic lipid levels (19). Of note, the relation of kATP and IHL largely varied with the absolute amount of IHL (Fig. 3B). It has previously been shown that reduced ATP synthesis in the liver is associated with IHL increase and a progression from simple steatosis to steatohepatitis (17). Moreover, an adaptive increase in hepatic mitochondrial lipid oxidation was observed in patients with early stages of NAFLD, which was consequently lost with liver disease progression (34). Based on the results from our study we assume that the proposed shift in hepatic energy turnover might occur at a very early stage of NAFLD, since we observed a positive correlation between IHL and kATP only in individuals with low normal IHL.

Analyzing hepatic phosphor metabolites, we found significant differences between the control group and NAFLD in PME, PDE, some of their individual components, and in NAPDH (see Table 1). PME and PDE are considered as cell membrane precursors and cell membrane degradation products, respectively (26). NADPH is an important factor for lipid and nucleotide synthesis as well as for defense against oxidant stress (35, 36). Previous studies in liver cirrhosis reported increasing levels of PME and decreasing levels of PDE with progressing liver steatosis and fibrosis (26, 37, 38). Increased levels of NADPH were associated with higher levels of ballooning in NAFLD patients (26, 37). However, our results show lower PME/PDE ratios and higher NADPH levels in the NAFLD group. As we included participants with a very low likelihood of fibrosis based on their FIB-4 scores, our findings might suggest that—similar to kATP—PME/PDE and NADPH might be markers of disease severity in NAFLD. They might initially adapt with increasing IHL up to a certain threshold and then inverse in progressed hepatic steatosis and fibrosis. These adaptive changes could be at least partially mediated by GH/IGF-I activity.

Some limitations of our study need to be addressed. Only otherwise metabolically healthy individuals without diabetes, severe obesity, and with a low FIB-4 score were included to avoid heterogeneity. Therefore, the impact of GH/IGF-I axis on hepatic energy metabolism in advanced liver disease is unknown. The whole study population was equally split between the sexes. However, significantly more men were in the NAFLD group. As expected, women had higher fasting GH as well as a higher nadir GH and GH-AUC, which is probably due to effects of estrogen on increasing binding globulin concentrations, but also sex-specific differences in GH secretion might be relevant (39, 40). With regards to methodological issues, noninvasive laboratory indices, but no results of liver biopsies, were available to rule out advanced hepatic fibrosis. Finally, disorders in GH secretion were defined by an IGF-I concentration within the assay-specific limits of normal for sex and age, but not by stimulation testing, which might have been more sensitive to rule out GH deficiency (41). However, only participants without any history or clinical symptoms of pituitary disorders were included in this study

In conclusion, we demonstrate that the GH/IGF-I axis activity might have a strong impact on hepatic fat, lipid composition, and phosphate metabolism in otherwise metabolically healthy individuals without disorders in GH secretion. Dynamic GH concentrations during the OGTT were significantly lower in the NAFLD group and correlated strongly with IHL as well as with UI. GH and IGF-I both predict IHL accumulation independent of obesity and insulin resistance. Furthermore, phosphor metabolites in the liver, which are strongly associated with GH/IGF-I axis activity, could give a new insight into early detection of progressing liver steatosis. Based on these results, we assume that pharmacologic agents directly targeting the liver-specific effects of GH could offer a promising therapy for NAFLD and warrant further investigations.

## Data Availability

Some or all data sets generated during and/or analyzed during the present study are not publicly available but are available from the corresponding author on reasonable request.

## Abbreviations

ATP: adenosine triphosphate
AUC: area under the curve
FIB-4: fibrosis-4
GH: growth hormone
GPC: glycerophosphocholine
HOMA-IR: Homeostasis Model Assessment of Insulin Resistance
IGF: insulin-like growth factor
IHL: intrahepatic lipid
MRS: magnetic resonance spectroscopy
NADPH: nicotinamide adenine dinucleotide phosphate
NAFLD: nonalcoholic fatty liver disease
OGIS: Oral Glucose Insulin Sensitivity
OGTT: oral glucose tolerance test
PDE: phosphodiester
PME: phosphomonoester
UDPG: uridine diphosphate glucose
UI: unsaturation index
ULN: upper limit of normal
